# Winner of the 2026 Tang Youqi Award

**DOI:** 10.3390/molecules31203704

**Published:** 2026-10-16

**Authors:** Zhongfan Liu, Chunying Chen, Xuesi Chen, Xiaoming Feng, Zijian Guo, Chunming Xu

**Affiliations:** 1 College of Chemistry and Molecular Engineering, Peking University, Beijing 100871, China; 2 Beijing Graphene Institute, Beijing 100095, China; 3 National Center for Nanoscience and Technology, Beijing 100190, China; chenchy@nanoctr.cn; 4 Changchun Institute of Applied Chemistry, Chinese Academy of Sciences, Changchun 130022, China; xschen@ciac.ac.cn; 5 College of Chemistry, Sichuan University, Chengdu 610064, China; xmfeng@scu.edu.cn; 6 School of Chemistry and Chemical Engineering, Nanjing University, Nanjing 210023, China; zguo@nju.edu.cn; 7 State Key Laboratory of Heavy Oil Processing, China University of Petroleum, Beijing 102249, China; xcm@cup.edu.cn

Professor Tang Youqi was a distinguished Chinese physical chemist and educator and one of the pioneers of structural chemistry and crystal chemistry in China. His scientific contributions played an important role in establishing crystal chemistry as a discipline in China. Among his major achievements, he proposed the theory of spontaneous monolayer dispersion on solid supports and introduced the concept of molecular engineering to guide the rational design of functional materials and catalysts [1]. He also led research on the determination of the crystal structure of porcine insulin, contributing to the early development of structural biology and biomacromolecular research in China. The concept of spontaneous monolayer dispersion was subsequently applied to the development of high-efficiency adsorbents and industrial catalysts, with applications in petrochemical and separation technologies. In recognition of his contributions to crystal chemistry, catalysis, and related areas of chemical science, Professor Tang received numerous national scientific honors. He was elected a Member of the Chinese Academy of Sciences in 1980 and served as Vice-President of the International Union of Crystallography from 1987 to 1990 [2].

The Tang Youqi Award was established by MDPI in 2025 to honor Professor Tang Youqi’s scientific legacy and to encourage continued innovation among younger generations of researchers in chemistry. The Award is presented annually to a researcher who has made significant and innovative contributions to fundamental research or the translation of scientific discoveries in chemistry and chemical engineering.

Nominations for the inaugural Tang Youqi Award closed on 10 April 2026. Following a rigorous evaluation process by the Award Committee, Professor Hailin Peng of the College of Chemistry and Molecular Engineering at Peking University was selected as the recipient of the 2026 Tang Youqi Award.

Professor Peng received his bachelor’s degree from Jilin University in 2000 and his Ph.D. from Peking University in 2005. From 2005 to 2009, he conducted postdoctoral research at Stanford University, after which he joined Peking University. His research focuses on the physical chemistry of two-dimensional (2D) materials and their applications in next-generation electronic and semiconductor technologies.

Professor Peng has made a series of contributions to the development, fundamental understanding, and large-scale integration of 2D electronic materials. His research has advanced bismuth-based 2D materials and prototype transistor devices with performance exceeding that of conventional silicon-based counterparts. His work has also contributed to the understanding of quantum phenomena in 2D systems, including AB quantum interference and the even-integer quantum Hall effect through surface and interface modulation. In parallel, he and his collaborators have advanced the wafer-scale fabrication and integration of 2D electronic materials and developed related technologies and equipment for their application in future electronic devices.

Beyond these fundamental advances, Professor Peng’s research has contributed to the translation of 2D materials into practical technologies. He has published more than 300 scientific papers, authored three monographs in Chinese, and been granted more than 90 patents. His research achievements have been recognized among the “Top 10 Advances in China’s Chip Science” in 2023 and the “Top 10 Research Advances in Semiconductors of China” in 2023 and 2025. He has also contributed to the commercialization of technologies including graphene wafers and electron microscopy grids, with related products included in the “List of 100 New Technologies and Products” presented at the Zhongguancun Forum—International Technology Exchange Conference. Selected publications by Professor Peng and his collaborators are provided in the bibliography of this Editorial [3,4,5,6,7,8,9,10,11,12].

In summary, it has been an honor for us to serve as members of the 2026 Tang Youqi Award Committee. It is our collective view that Professor Hailin Peng’s sustained contributions to the physical chemistry of two-dimensional materials, together with his advances in semiconductor materials, device technologies, and the translation of fundamental research into practical applications, make him a highly deserving recipient of the inaugural Tang Youqi Award.
